# Untested, unproven, and unethical: the promotion and provision of autologous stem cell therapies in Australia

**DOI:** 10.1186/scrt543

**Published:** 2015-02-09

**Authors:** Alison K McLean, Cameron Stewart, Ian Kerridge

**Affiliations:** Sydney Medical School, University of Sydney, Edward Ford Building (A27), Fisher Road, Sydney, NSW 2206 Australia; Centre for Values, Ethics and the Law in Medicine, K25, Medical Foundation Building, Sydney Medical School, University of Sydney, 92-94 Parramatta Road, Camperdown, NSW 2006 Australia; Haematology Department, Royal North Shore Hospital, St Leonards, Sydney, NSW 2065 Australia; Northern Blood Research Centre, Kolling Institute, Reserve Road, St Leonards, Sydney, NSW 2065 Australia

## Abstract

An increasing number of private clinics in Australia are marketing and providing autologous stem cell therapies to patients. Although advocates point to the importance of medical innovation and the primacy of patient choice, these arguments are unconvincing. First, it is a stark truth that these clinics are flourishing while the efficacy and safety of autologous stem cell therapies, outside of established indications for hematopioetic stem cell transplantation, are yet to be shown. Second, few of these therapies are offered within clinical trials. Third, patients with chronic and debilitating illnesses, who are often the ones who take up these therapies, incur significant financial burdens in the expectation of benefiting from these treatments. Finally, the provision of these stem cell therapies does not follow the established pathways for legitimate medical advancement. We argue that greater regulatory oversight and professional action are necessary to protect vulnerable patients and that at this time the provision of unproven stem cell therapies outside of clinical trials is unethical.

## Introduction

Currently, a number of private clinics in Australia are advertising and selling stem cell therapies to patients. These therapies are largely untested and unproven and the results of these treatments have not been published in the scientific literature. As these therapies are generally not subsidized by the government or by private insurers, vulnerable patients who often have few other options are consequently assuming the not insignificant costs and the uncertain risks of these treatments in the absence of clear evidence of their efficacy.

We argue that these practices need greater regulatory scrutiny. This article begins with a brief examination of current stem cell science and what we know about stem cell therapy. We then describe the range and characteristics of autologous cellular therapies being offered in Australia. The article examines the existing regulation of these therapies (including therapeutic goods law, professional discipline, and consumer law) and the ethics of innovative therapies. We conclude that the current practice of offering unproven cellular therapies outside of clinical trials is unethical and that much more could be done to better regulate this practice.

## The current science of stem cell therapy

Although stem cell therapies are likely to become an increasingly used therapeutic modality in skin grafting and corneal repair, the only adult stem cell therapy that is currently accepted for therapeutic use as standard best practice is hematopoietic stem cell transplantation (HSCT) [1]. Autologous and allogeneic HSCT is used to treat a wide range of hematological malignancies, autoimmune diseases, and genetic conditions (Table 1) [2].

**Table 1:** **Diseases commonly treated with hematopoietic stem-cell transplantation**

	Autologous transplantation	Allogeneic transplantation
Malignancies	Multiple myeloma	Acute myeloid leukemia
	Non-Hodgkin’s lymphoma	Acute lymphoblastic leukemia
	Hodgkin’s disease	Chronic myeloid leukemia
	Acute myeloid leukemia	Myelodysplastic syndromes
	Neuroblastoma	Myeloproliferative disorders
	Ovarian cancer	Non-Hodgkin’s lymphoma
	Germ-cell tumors	Hodgkin’s disease
		Chronic lymphocytic leukemia
		Multiple myeloma
		Juvenile chronic myeloid leukemia
Other diseases	Autoimmune disorders	Aplastic anemia
	Amyloidosis	Paroxysmal nocturnal hemaglobinuria
		Fanconi’s anemia
		Black-fan diamond anemia
		Thalassemia major
		Sickle cell anemia
		Severe combined immunodeficiency
		Wiskott-Aldrich syndrome
		Inborn errors of metabolism

Adapted from Copelan [2] (2006).

Adult hematopoietic and non-hematopoietic stem cells are also being investigated in preliminary clinical trials for the treatment of a range of diseases. A number of preliminary trials have been published investigating the use of stem cells to improve heart function [3], stroke [4], peripheral arterial disease [5], and amyotrophic lateral sclerosis [6]. A phase II trial under way in Australia is investigating autologous HSCT for multiple sclerosis (MS) [7], and mature results of the trial are not expected for many years. A non-randomized pilot study of nine patients who received intra-articular injections of autologous adipose-derived mesenchymal stem cells (MSCs) for osteoarthritis (OA) showed improvement in pain and in arthroscopic, histological, and radiological outcomes [8]. However, this pilot study lacked a control group, which is crucial to demonstrating the efficacy of such treatments as there is a clearly demonstrated placebo effect in the treatment of OA [9]. Importantly, no randomized, double-blinded, multicenter clinical trials of sufficient statistical power have been published that provide generalizable clinical data demonstrating the efficacy of adult stem cell therapies over currently available standard practice [10].

Although the use of MSCs appears to be safe, further evaluation with large-scale clinical trials is required to determine the safety profile [11]. A number of risks are known to be associated with autologous stem cell therapies, including thrombosis, acute lung injury, infection, benign tumor growth, and malignant transformation, and many more things remain unknown [12, 13]. Three recent case reports highlight the uncertain risks of stem cell therapy. In the first, a boy who received an intrathecal injection of human fetal stem cells developed a brain tumor derived from the transplanted neural cells 4 years after treatment [14]. In the second, a patient who received an olfactory mucosal cell transplantation at the site of spinal cord transection presented with a spinal cord mass from the transplanted cells 8 years later [15]. Third, a patient undergoing autologous stem cell therapy for lupus nephritis by direct renal injection developed angiomyeloproliferative mass lesions at the site of injection [16]. Additionally, serious adverse effects after administration of autologous cells by intravenous and intracardiac routes and into the central nervous system have been reported [17–19].

## The practice of private stem cell clinics in Australia

Even though stem cell science is at a relatively early stage and there are limited mature clinical data, adult stem cell therapies are already being provided directly to patients in private clinics across Australia. We identified 17 private clinics and three stem cell companies that currently provide autologous adult stem cell therapies. These clinics offer autologous stem cell therapies for a wide range of diseases outside of clinical trials, including OA and musculoskeletal pain; neurodegenerative disorders such as muscular dystrophy and MS; stroke; retinal neuropathy; spinal cord injury; headache and migraine; asthma; autism; ‘facial rejuvenation’; and anti-aging. The medical professionals who provide stem cell therapies in these private clinics in Australia include cosmetic surgeons, sports medicine physicians, orthopedic surgeons, and general practitioners, and specialists in the diseases being treated in these clinics, such as neurologists, generally are not involved in the provision of care. Most of these clinics have websites with detailed information regarding treatments available, media coverage of the clinic, health professional profiles, and the cost of treatment.

### A lack of reliable research

Seven of these clinics claim to participate in research regarding stem cell therapies. However, with one exception, the health professionals at these clinics appear not to have published their findings in peer-reviewed journals. One publication (which lacked statistical analysis or evidence of ethics approval and was published in a journal not indexed by Medline) described a ‘pilot study’ involving six patients whose OA was treated at the clinic [20]. In this regard, it is noteworthy that the clinic claims to have provided treatment to more than 300 patients [21].

A blinded randomized controlled clinical trial of 40 patients with OA used technology provided by one of the stem cell companies, but results are yet to be published, although the trial status is listed as closed with follow-up complete. The company released an interim report of the results of this clinical trial, stating that stem cell therapy had reduced pain scores in the treatment arm; however, the results of the trial showed no difference in the reduction of pain scores between the treatment and placebo arms of the trial [22].

### The use of narratives in advertising

The information available on the clinics’ websites commonly includes patient and doctor narratives describing the ‘dramatic’ effect of autologous stem cell therapy. Seven clinics provide selected patient testimonials, presenting only those patients who have undergone ‘successful’ stem cell treatments. Patients from four clinics, along with their doctor, have appeared on local Australian current affairs television programs to describe the benefits they have experienced following stem cell treatment [23–27]. Celebrity patients, in particular, have featured prominently in television reports of stem cell therapies, including a television presenter and model, a rugby league player, a retired Australian test cricket bowler, and a former Olympic volleyball player. Social media has also been used to support individual patients ‘access’ to stem cell therapies, and one clinic allowed a general practitioner to provide autologous stem cell therapy to an 8-year-old boy with autism after his parents used social media to raise the necessary funds [28].

## Unsupported claims

On their websites and in media interviews, both the clinics and the health professionals who provide stem cell therapies frequently make claims about efficacy and safety that cannot be supported by published evidence, and any support for the veracity of these claims rests solely on local, unpublished data collected at their own clinic. One clinic advertises that stem cell therapy is a ‘proven’ therapy for joint pain [29] and other clinics state that stem cell therapy is reliably effective [30] and ‘appears 100% safe’ [31]—a claim that is inconsistent with the possibility of infection and the small possibility of malignancy reported with stem cell therapies [32]. Three clinics also claim 80% to 90% efficacy of stem cell therapy and no way of predicting those patients who are ‘non-responders’ to treatment [21, 33, 34]. Yet another clinic claims that stem cell therapy can improve the strength, energy, and stamina of patients with muscular dystrophy [35] and relieve migraine in 98% to 99% of patients [35]. One company claims that a technology it supplies can generate pluripotent stem cells from the patient’s peripheral blood [36].

## Costs of treatment

Autologous stem cell therapies, without exception, are expensive. The cost of intra-articular injection of stem cell is at least $9,000 (excluding the costs of consultation by the health professionals, cell storage, and subsequent therapy). The cost of stem cell therapy for autism is $12,000 [37]. The cost of cosmetic injections of stem cell therapy varies from $750 to $2,500 depending on the treatment. Three clinics offer payment plans for between $60,000 and $70,000 which can be repaid over the course of 84 months. In all cases, these costs are not reimbursed by Medicare, the financial scheme that covers the costs of approved medical care in Australia.

## The regulatory context in Australia

In Australia, the regulation of therapeutic goods, including prescription, non-prescription, and complementary medicines as well as medical devices, is overseen by the Therapeutic Goods Administration (TGA) [38]. The TGA regulates cell products, including blood products, vaccines, and hematopoietic stem cells, used in allogeneic transplantation. However, the TGA has specifically excluded from its regulatory jurisdiction human cells that are collected from a patient who is under the clinical care and treatment of a registered medical practitioner if the cells are manufactured by that medical practitioner for therapeutic application in a single treatment [39]. Consequently, any registered medical practitioners in Australia can offer autologous stem cell therapy to patients for a single treatment or disease, such as OA, completely outside of any form of regulation by the TGA.

At the same time, autologous stem cell therapies are regulated by the same mechanisms that regulate clinical practice, including the Medical Board of Australia and the Australian Health Practitioner Regulation Agency (AHPRA) in accordance with the Health Practitioner Regulation National Law [40]. The Medical Board of Australia produces standardized guidelines and codes of conduct for good clinical practice [40]. The AHPRA and its co-regulatory partners investigate allegations of unprofessional conduct and poor performance. Sanctions may be imposed on practitioners, such as suspending medical registration, and fines may be imposed on health professionals found guilty of professional misconduct or unsatisfactory professional conduct [41]. In this regard, it is noteworthy that, as of June 2014, three health practitioners have been disciplined for providing stem cell therapies, and one had his medical registration suspended for three years. (In *Medical Board of Queensland v Tarvydas* (2010) QCAT 246, a doctor was found guilty of unsatisfactory professional conduct, had his registration cancelled, and was prevented from reapplying for registration for 3 years for offering stem therapy for adhesive arachnoiditis. Other health practitioners such as chiropractors (Chiropractic Board of Australia v Hooper (Review and Regulation) (2013) VCAT 878) and a Chinese health specialist (Chinese Medicine Registration Board of Victoria v Ghaffurian (Occupational and Business Regulation) (2012) VCAT 478) have also been disciplined for offering stem cell-related therapies.)

The common law also provides a regulatory framework through actions in negligence which can be brought by patients who have suffered damage as a result of the medical care which is not supported by peer professional opinion as being competent professional practice [42]. Australian consumer law may also have some role to play, as businesses are not allowed to make false or misleading statements or representations regarding the quality and standard of products either to the consumer or through advertising or promotional material [43, 44]. For example, if a business predicts the health benefits of a therapeutic device or health product but has no evidence that such health benefits can be attained, this would constitute misleading conduct [45]. It seems, therefore, that the regulation of these therapies is weak and the regulatory mechanisms available are primarily reactive, rather than proactive, as they require complaints to be made and investigated before the systems will respond.

## Ethics of innovative therapies

Medical therapies become an accepted part of standard medical practice through two different pathways, either following rigorous evaluation through research or following innovation occurring in the course of clinical practice. The distinction between standard therapy, research, and innovative therapies is important as they attract different regulatory frameworks.

Standard therapies are those therapies that have been sufficiently tested and accepted by peer review or relevant regulatory bodies [46]. Research is a systematic investigation to establish facts, principles, or knowledge and a study of some matter with the objective of obtaining or confirming knowledge [47]. There are two central aspects to the definition of innovative therapies: (a) the departure from standard medical therapy and (b) that the therapy has not been validated by reliable research methods or there is not enough available evidence to support the safety and efficiency of the therapy required for acceptance or approval from peers and regulatory bodies [46, 48, 49]. In regard to both standard clinical practice and innovative therapies, patient care is paramount, whereas in the research setting, although close attention is still paid to protecting patients and promoting their welfare, emphasis is also paid to generating new knowledge through research with populations of patients who may or may not receive therapies that subsequently turn out to have little or no benefit and that may be associated with significant harms [48].

Innovation is a useful and legitimate tool for advancing the body of medical knowledge [46]. Many of the major advances in surgery that are considered routine today, such as laparoscopic surgery, were introduced through innovation, with little regulatory oversight, rather than through research [50–52]. Importantly, in Australia, the introduction of new innovative surgical procedures is not integrated into any regulatory framework as it falls outside the ambit of major regulatory bodies such as the TGA [53].

Innovative practice is currently regulated as clinical practice through codes of conduct, internal review, disciplinary and investigational bodies such as AHPRA, and medical negligence law. One concern regarding innovative therapy is that it creates a potential conflict between the patient’s best interests and the personal, professional, and financial interests of the physician [48, 54, 55]. A direct conflict of interest arises if the physician has a vested financial or non-pecuniary interest in demonstrating the success of the innovative therapy because of, for example, a financial stake in the intellectual property connected to the innovative therapy or a professional or academic interest in the success of the innovation [48, 49]. It is arguable that, for this and other reasons, greater regulatory oversight is required to protect the best interests of patients and the community.

The International Society for Stem Cell Research (ISSCR) supports innovative stem cell therapies and provides a series of recommendations to clinicians implementing innovative stem cell therapies. The ISSCR recommends a number of extra criteria that should be satisfied in order to legitimately use stem cells in the context of innovation, such as a written protocol which outlines the scientific rationale for the use of stem cells and formal follow-up of patients. According to the ISSCR Guidelines, such innovative therapies should be supported by clinical and administrative leadership, be carried out by appropriately qualified personnel, ensure that patients give voluntary informed consent, provide an action plan for adverse events, be appropriately insured, and be provided as part of a commitment by the researchers and scientists toward the generation and dissemination of generalizable knowledge [56]. The additional requirements placed on innovative therapies involving stem cells by the ISSCR are necessary, as therapeutic stem cells and their derivatives can vary in source, manufacture, processing, potency, storage, and route of delivery and have special risks such as tumorgenicity and permanent integration into recipients, and the long-term activity of stem cells also requires that there be long-term follow-up of patients [49].

## Conclusion

Despite the lack of evidence for the use of stem cell therapies in humans other than in the context of hematopoietic transplantation, at least 17 private clinics across Australia provide autologous stem cell therapies for a wide range of diseases [57]. Most of these clinics have sophisticated websites that create the impressions that the therapy is evidence-based and rests on rigorous scientific and clinical data [10]. For example, the websites include the following: lists of conferences attended by professionals; claims of having patented technologies that do not appear to exist in the Australia patent database; explicit claims that stem cell therapies are effective along with disclaimers that the treatment is ‘experimental’ and outcomes may vary; references to clinical trials that have not progressed in over 3 years and to ongoing clinical trials that are not registered, by another clinic; claims that clinics are authoritative and ‘expert’ because the health professionals who work there are involved in writing a code of conduct for stem cell therapies in consultation with government and universities [58]; and widespread use of supportive patient testimonials and media reports regarding stem cell technologies and omission of any reference to more circumspect publications about autologous stem cell therapies, such as those produced by the National Health and Medical Research Council (NHMRC) and the ISSCR [1]. What is striking about these websites, therefore, is the way in which they use speculation, anecdote, media reports, and patient narrative instead of scientific evidence to promote stem cell therapies [10].

The use of patient testimonials is particularly noteworthy because testimonials have been shown to outweigh prospective patient concerns regarding the risks of stem cell therapies [59]. The use of patient testimonial to advertise, market, and promote autologous stem cell therapies also arguably contravenes Australian law and related guidelines regarding the advertising of health services [40, 60–62].

The lack of published data about these treatments is extremely concerning. Medical practitioners working at these stem cell clinics have provided treatment to hundreds of patients over a number of years but have not published the findings in peer-reviewed journals listed on PubMed. The lack of publications suggests both a limited commitment to research (as opposed to commerce) and a lack of openness and transparency in communicating methods and results with the medical community [63]. The high success rates of stem cell therapy stated on the websites of stem cell clinics and shared in media interviews may create a misconception among patients that the procedure is standard clinical practice [64]. In reality, of course, these claims are based upon a pathophysiological rationale that is rarely made explicit and upon local ‘data’ that have not been verified through peer review or publication. We believe that the claims set out by practitioners create an unreasonable expectation of the safety and efficacy of stem cell therapies, which therefore could be considered in breach of the National Law as well as health-related advertising standards in Australia [40, 60, 65–67].

Given the lack of evidence of the efficacy of stem cell therapies [1, 10], the cost of these therapies is exorbitant. The charges that patients pay are even more difficult to justify given that the medical practitioners involved stand to gain financially and professionally by promoting and selling stem cell therapies directly to patients.

Innovation in clinical practice should, of course, be encouraged. However, autologous stem cell therapies provided to hundreds of patients in private clinics across Australia rarely can be considered innovative therapies. Innovative therapies should be limited to a small number of ill patients who have no other alternatives [56, 63, 68]. Innovative therapies should also be individualized to each patient [63]. The fact that patients can go up to $70,000 in debt through a single clinic further suggests that the clinics are large-scale commercial operations and that they do not provide innovative therapies [68, 69].

Likewise, the clinics that are offering autologous stem cell therapies outside the context of established hematopoietic transplantation are generally not engaged in research or in building databases that may contribute to the meaningful assessment of their efficacy. In general, the provision of these therapies is not based upon an identifiable research question or hypothesis, nor approval by a research ethics committee to conduct research on human subjects. There is also no responsible dissemination of research findings through publication.

We suggest that the large-scale provision of unproven stem cell therapies outside of clinical trials be prevented through appropriate regulation or oversight and that legitimate innovation be encouraged and not hampered by over-regulation. Furthermore, clinical innovation in autologous stem cell therapies should be subject to scientific and ethical review and have built-in patient protection [56, 68].

Allowing the provision of unproven autologous stem cell therapies to continue outside of clinical trials is enormously problematic [55, 69–71]. Firstly, these patients are exposed to infrequent but still largely unknown risks. Secondly, the patients who receive these treatments may be excluded from participation in clinical trials because of the unknown effects of unproven stem cell therapies [69]. Thirdly, undergoing stem cell therapy may lead to a delay for patients seeking proven beneficial therapies [69]. Finally, the manner in which these clinics advertise and provide unproven therapies arguably undermines the integrity of the medical profession and the research community [69] and the successful translation of stem cell research into clinical practice [63].

Greater oversight of autologous stem cell therapies in Australia is required. A number of strategies could be pursued. One option is for the TGA to repeal its exclusion, so that autologous stem cell therapies would attract the same regulatory safeguards as other therapeutic goods. Policing of unsubstantiated claims on websites and media appearances through consumer laws would help to reduce the misconception that unproven stem cell therapies are safe, efficacious, and established treatments. Providing education and information for health professionals about the realistic expectations about stem cell therapies and the existence of clinics providing unproven and expensive stem cell therapies (as the NHMRC has already done) may enable patients to make better-informed decisions about these therapies [55]. In particular, patients should be cautioned that just because a stem cell therapy is offered in Australia does not make it legitimate [55]. Education and information should also be targeted at patients to empower them to take responsibility for their health choices. However, the information provided needs to reach a wider public audience and so should harness the same media platforms as the clinics themselves. A register of innovative stem cell therapies might also provide a mechanism to assess new stem cell therapies by evaluating the scientific and ethical rationale before a clinician has the opportunity to provide treatment to hundreds of patients without oversight and could facilitate both the long-term follow-up of patients and distinction of legitimate from illegitimate innovation. The medical profession itself should also take responsibility to ensure that practitioners clearly stepping outside the bounds of ethical and responsible clinical practice be properly and publicly reprimanded through the professional regulator (AHPRA), so that situations in which cosmetic surgeons provide treatment to patients with amyotrophic lateral sclerosis, OA, and asthma simply do not occur.

Individuals suffering from incurable conditions will often pursue any chance of hope. Private stem cell clinics across Australia have been allowed to take advantage of this vulnerable patient population because of the absence of adequate regulation and oversight and the apparent unwillingness of regulatory bodies and research and health professionals to act on existing breaches of practice [55, 69]. Greater oversight and greater action are necessary to prevent the exploitation of desperate patients, to protect the probity of the medical profession, and to limit the cost to society as a whole.

## Authors’ information

AKM is affiliated with the Sydney Medical School of the University of Sydney (Sydney, Australia). CS is professor of law and pro dean of the Faculty of Law at the University of Sydney and is affiliated with the Centre for Values, Ethics and the Law in Medicine at the School of Public Health at Sydney Medical School. IK is hematologist/bone marrow transplant physician at Royal North Shore Hospital in Sydney and director and associate professor of bioethics at the Centre for Values, Ethics and the Law in Medicine.

## Abbreviations

AHPRA: Australian Health Practitioner Regulation Agency
HSCT: Hematopoietic stem cell transplantation
ISSCR: International Society for Stem Cell Research
MS: Multiple sclerosis
MSC: Mesenchymal stem cell
NHMRC: National Health and Medical Research Council
OA: Osteoarthritis
TGA: Therapeutic Goods Administration.
